# Self and collective care for faculty and staff: A pilot intervention promoting well-being in a nursing education setting

**DOI:** 10.1097/nmg.0000000000000280

**Published:** 2025-07-31

**Authors:** Elisabeth Bailey, Dar'ya Dyachuk

**Affiliations:** At the University of British Columbia in Vancouver, British Columbia, Canada, **Elisabeth Bailey** is an Associate Professor of Teaching and the Associate Director, Undergraduate Programs, School of Nursing, Faculty of Applied Science; and **Dar'ya Dyachuk** is a Research Assistant, LEaRN Lab, Faculty of Applied Science.

**Figure:**
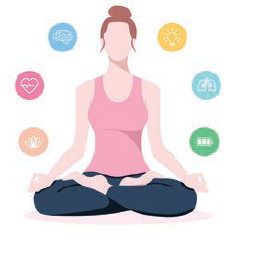
No caption available.

**PROGRAMS TO ENHANCE** nurses' well-being have flourished in recent years, with a surge in offerings during the COVID-19 pandemic.^1^-^3^ Evidence suggests that these initiatives benefit nurses by enhancing social connection, bolstering coping skills, and helping individuals identify effective self-care strategies.^1^,^3^ However, studies have also demonstrated that employee wellness programs vary in effectiveness, and those that utilize individual-level interventions often fail to significantly improve key indicators of personal and professional well-being, such as self-reported mental health, regular physical activity, and frequency of absenteeism.^4^,^5^ Investments in nurses' well-being, therefore, must be directed toward strategies with proven efficacy and a focus on both individual and collective well-being.

## BACKGROUND

Like their colleagues in practice, nursing students and educators are navigating a stressful postpandemic environment. Shortages of nurses in both clinical settings and academia, along with limited availability of practice sites, put all involved in nursing education at a risk for burnout.^2^,^6^-^8^

Numerous studies have demonstrated that nursing students face multifaceted stressors.^7^ As Sharpnack describes, the “intensive pressure and rigor of nursing education, the emphasis on acquiring the requisite knowledge and skills to practice safely, and the gravity of clinical experiences contribute to stress and burnout among nursing students.”^9^ Similarly, nursing faculty members are reporting heightened levels of exhaustion and burnout largely attributed to increased workloads and difficulties maintaining work-life balance, challenges that contribute to a growing shortage of nurse educators.^6^,^8^,^9^

Acknowledging the stress inherent in nursing education, our school of nursing developed the Self and Collective Care Series (the series), a set of four modules designed to support undergraduate nursing students to gain skills related to self-regulation and coping, reflective practice, social belonging, and collective care. Each of the four modules features an asynchronous learning component and a 60-minute facilitated debrief session delivered in small groups of 8 to 10 students, either in person or via Zoom. The modules are intentionally designed to progress from an individualized approach to self-care in the first module, to a deeper exploration of the limits of individualism and the importance of collective care and systems-level supports in the final module. See Table 1 for an overview of topics and content covered in each module.

**TABLE 1:** Self and Collective Care Series of modules

Topic	Content
Module 1: Understanding Stress, Resilience, and Self-Care	Why does self and collective care matter in nursing? Exploring domains of well-being and the stress cycle Building an effective self-care plan
Module 2: Self-Care – In-the-Moment Strategies	Understanding the stress response and “resilient zone” Evidence-based strategies to support self-regulation in stressful situations
Module 3: Self-Care – Strategies to Promote Wellbeing	Evidence-based strategies to support day-to-day well-being Mindfulness, gratitude, and rituals Social connection
Module 4: From Self to Collective Care	The limits of individual resilience and self-care – organizational and systemic responsibilities The plan going forward – transition to practice

## UNDERSTANDING “SELF AND COLLECTIVE CARE”

In the series, we understand self-care as “a broad range of activities and practices...undertaken to promote and protect one's health and well-being and to cope with and build capacities for managing self-identified stressors.”^10^ With respect to collective care, we look to Doane and Varcoe's work on relational inquiry, which explains that “patient well-being, nurse well-being, and health care system well-being are intricately interwoven. These three concerns are inherently related and dependent on one another...Thus, specific attention to all three of these elements is necessary for effective nursing action.”^11^

Doane and Varcoe argue that when nurses are supported to engage authentically and ethically with patients and colleagues, nurse well-being is enhanced. Well-supported nurses, in turn, positively affect patient well-being. Doane, Varcoe, and colleagues also stress that systemic structures, such as organizational policies, leadership practices, and resource allocation, must be aligned to sustain both nurse and patient well-being.^11^,^12^ Thus, improvement efforts focused on one aspect—nurse, patient, or healthcare system—require attention to the interactions of the whole relational system. In the self and collective care work that we present in this article, we take into consideration the nurse-patient-healthcare system relational whole, as well as the parallel—and we suggest comparable—triad of nursing educator, nursing student, and nursing education system.

### Intervention

This article presents findings from a pilot project designed to enhance the individual and collective well-being of faculty and staff at a school of nursing in Western Canada. Our project team adapted the four modules of the series described above into a resource for faculty and staff. The adaptation involved reviewing and revising student modules for relevance to faculty and staff and modifying synchronous, facilitated discussions to meet the needs of a group of colleagues versus student peer groups.

Project goals included introducing faculty and staff participants to the content, resources, and strategies that students explore in the original version of the series, while offering participants opportunities to reflect on how self and collective care fit into their personal and professional lives. A long-term goal is building a common language, understanding, and organizational culture supportive of self and collective care.

## METHODS

### Design and implementation

Using the series of modules created for nursing students as a foundation, this pilot project involved adapting the modules to be relevant for faculty and staff. Adaptation included adding contextual notes to asynchronous learning materials to help faculty and staff participants understand when and where students encountered the elements of the series in their education. Prompts were also added, specific to participants' roles as faculty or staff members (for example, “How does your personal relationship to self-care impact your role as a nurse, a teacher, and/or a colleague or leader? What can you effectively role model for students and colleagues?”).

Participants reviewed the asynchronous learning module independently (approximately 1 to 2 hours to complete), and, after each asynchronous module, came together for a facilitated discussion. One coauthor (EB) facilitated each session and followed a set agenda.

- Welcome and two-word check-in (such as what two words describe how you are arriving today?)
- Brief guided meditation
- Group discussion based on prompts:
  – What were your main takeaways from the asynchronous module?
  – How have you been enacting self-care? If you haven't been successful at enacting elements of your self-care plan, what do you think would help to move forward?
- Sharing an intention for the week ahead

The agenda was intentionally structured to enhance opportunities for connection among participants and to reinforce strategies introduced in the asynchronous modules. Research on psychological safety in the workplace indicates that team rituals support rapport building and shared accountability.^13^ Starting each facilitated session with a two-word check-in and ending with intention-setting bookended the sessions with rituals of sharing, while also normalizing conversations about emotions, areas of struggle, and opportunities for personal and professional growth. Drawing on contemplative pedagogic approaches, a brief mindfulness meditation was included in each session with the goal of cultivating focus and attention before diving into discussions.^14^ Incorporating this mindful meditation enabled participants to transition from the distractions of their day and fully attend to the discussion, while practicing an evidence-supported stress management strategy.^12^

Calendar invitations with links to the asynchronous modules and discussion prompts for consideration were shared with participants when they expressed interest in joining the pilot. Automatic reminders were sent a week before the scheduled synchronous discussions. Small group sessions were offered in a hybrid format; participants could join in-person or via Zoom. All sessions had a mix of in-person and virtual participation.

### Participants

Using a department email list, faculty and staff members were invited to participate in the Self and Collective Care Series: Faculty and Staff Adaptation. Fifteen individuals engaged with at least one aspect of the series (by accessing the asynchronous learning modules, attending a facilitated session, or both), eight participants completed the presurvey, and seven participants completed postsurveys after finishing the asynchronous modules and participating in facilitated group conversations.

### Ethical considerations

The project team consulted our university's Behavioral Ethics Review Board's published checklist for quality improvement and program evaluation projects and determined that ethics board approval wasn't required for this program evaluation. Participation in all aspects of this project was voluntary. There were no known conflicts of interest among participants and the facilitator, and participants could opt out at any time. Although participation in the facilitated group sessions couldn't be anonymous, pre- and postsurveys were completed anonymously.

### Program evaluation

Participants were invited to complete a presurvey, and feedback on the series was collected after the final facilitated group session via postsurvey. The pre- and postsurveys included queries about participants' knowledge, skills, and confidence related to self and collective care and incorporating self and collective care into their professional roles. Participants were also invited to share what they appreciated about the series and what suggestions for improvement they had, if any.

## RESULTS

### Survey results: Likert-type scale questions

The pre- and postsurveys asked faculty and staff to rate their knowledge, skills, and confidence related to self and collective care on a five-point scale before and after engaging with the series.

On presurveys, six out of eight participants rated self and collective care as extremely important, and the same number reported having no formal training related to these topics. Participants rated their knowledge as low to moderate (2-3 out of 5). Those who received formal training were more likely to rate their knowledge and confidence higher. Despite valuing self and collective care, most participants rated their confidence as neutral (5 of 8 participants) or low (2 of 8 participants) when it came to teaching these topics to nursing students.

Postsurvey results suggest that the series had a strong positive impact on participants' perceptions, confidence, and intent to apply what they learned into their nursing education practice. There was a notable increase in participants' knowledge and understanding of self and collective care and the relationship of self and collective care to their professional roles. Three participants rated their knowledge as very high (5 out of 5), and four participants rated it as high (4 out of 5). All participants found the content “very relevant” to their role as nursing faculty and/or staff, confirming that the material aligned well with their professional responsibilities and needs. All the participants also reported being “extremely likely” to apply the principles learned into their nursing education practice, suggesting a high level of engagement and motivation to integrate self and collective care into their own work with nursing students and colleagues.

### Survey results: Open-ended responses

Thematic analysis of the open-ended survey responses highlighted three themes: (1) reframing self-care as a priority, (2) integrating evidence-based strategies, and (3) building connections. Participants also described (4) facilitators of their learning and connection.

***1. Reframing self-care as a priority***. Engagement in the series highlighted that attention to self and collective care is crucial and that it must be supported organizationally. As one participant noted, “self and collective care is a critical part of our work, and not a ‘bonus’ activity. It needs to be prioritized individually, and as part of our workplace culture.”

Participants also appreciated the focus on those delivering nursing education noting, “I thought that it was a great initiative as we always stress self-care for students, so we must practice self-care ourselves as staff and teachers teaching them.”

***2. Integrating evidence-based strategies***. Participants in the series applied new strategies in their daily self-care practices; “[E]ach of the modules introduced me to new information and mental maps in which to consider my experiences. This brought new, practical tools to help guide choices and ways of being in my day.”

There was also direct application to teaching and supporting nursing students. One participant anticipated that they'd use “the specific tools/meditations and also more broadly the reminder/invitation to weave authenticity and vulnerability into my teaching.” Another noted that the strategies and practices introduced in the series would support them in creating “a safe and inclusive space to critically discuss and reflect our own personal and clinical experiences” with their learners.

***3. Building connections***. Participants appreciated the opportunity to connect with colleagues in a meaningful and supported way. They described the facilitated discussions as “a safe space for reflection on stress-management strategies and learnings and connection among colleagues,” and shared that “[i]t has been a validating experience and has also helped me to build new and stronger relationships with my colleagues.”

***4. Facilitators of learning and connection***. Although busy work schedules were noted to be a challenge, participants described several factors that facilitated their learning and connection to one another. The “flipped classroom” approach of asynchronous content coupled with facilitated discussion supported connection and reflection with colleagues, as well as opportunities for individuals to revisit materials as needed. The multimedia format of the asynchronous modules, which include embedded videos and voiceover of text, was engaging. Hybrid delivery allowed for more flexible participation.

Participants agreed that facilitation style was key to promoting psychological safety for the group, enabling participants to feel that it was a safe environment for interpersonal risk-taking.^15^ The facilitator should be skilled at creating an inclusive space that supports connection and more vulnerability than might be typical in professional contexts.

## DISCUSSION

Taken together, responses to Likert-type scale questions and open-ended survey responses indicate that the pilot offering of the series for faculty and staff was effective and impactful for participants. Presurvey responses indicated limited formal training, moderate knowledge, and neutral confidence levels, despite widespread agreement on the importance of self and collective care in nursing education. After completing the series, knowledge levels increased. Participants' confidence in promoting self and collective care also improved, with most participants shifting from neutral to confident or very confident in their ability to integrate learnings into their roles. All participants found the modules highly relevant to their roles, and all reported being extremely likely to apply what they learned into the professional practice. These results demonstrate the effectiveness of structured professional development in bridging knowledge gaps, boosting confidence, and encouraging application of self and collective care strategies in nursing education contexts.

## IMPLICATIONS FOR NURSE LEADERS

Key takeaways for nursing leaders, particularly in nursing education settings, are as follows:

- Effective initiatives to enhance well-being must go beyond a focus on individual resilience and intentionally attend to facilitating collective care and social support.
- Creating structured opportunities for individual learning about self-care, coupled with facilitated group conversations, fosters both individual skill-building and social connection.
- Creating psychologically safe spaces for collaboration and sharing is key. Sharing discussion prompts and session agendas ahead of time, making all contributions voluntary, and role modeling the opportunity to “pass” when responding to questions are strategies to foster a sense of safety among participants.
- Combining asynchronous and synchronous learning opportunities and including hybrid participation options for synchronous sessions increases engagement and perceived flexibility.

## LIMITATIONS

The small sample size of this pilot project is the primary limitation and impacts the ability to generalize efficacy and impact of this intervention beyond our specific organizational context. Findings from this pilot are promising, however, and warrant larger-scale delivery and assessment of the series.

## A VITAL INVESTMENT

A renewed focus on nurses' well-being is an important by-product of the COVID-19 pandemic. There are also increasing calls for concerted efforts to enhance the psychological safety of learning environments, given the importance of cultivating effective collaboration and a strong sense of accountability in new nurses and among healthcare teams.^16^

Nursing students are the future of healthcare delivery, and the educators and staff who support their training are critical to ensuring that the next generation of nurses are prepared to navigate the stress inherent in nursing practice. Our intervention offers an actionable approach to embedding evidence-informed, well-being—enhancing strategies into educational programs, better equipping both educators and future nurses for the complexities of teaching, learning, and practicing in healthcare.

Drawing from Doane and Varcoe's observations, we believe that faculty and staff well-being, student well-being, and nursing education system well-being are entwined and mutually reinforcing.^12^ Investing resources in promoting faculty and staff well-being supports the health and well-being of nursing students and nursing education systems. We believe this is a vital investment in a thriving future for nursing and healthcare.
